# Imaging in Transcatheter Aortic Valve Replacement (TAVR): role of the radiologist

**DOI:** 10.1007/s13244-013-0301-5

**Published:** 2014-01-21

**Authors:** Diana E. Litmanovich, Eduard Ghersin, David A. Burke, Jeffrey Popma, Maryam Shahrzad, Alexander A. Bankier

**Affiliations:** 1 Department of Radiology, Beth Israel Deaconess Medical Center, 330 Brookline Ave-Shapiro 4, Boston, MA 02215 USA; 2 Department of Radiology, Leonard M. Miller School of Medicine, University of Miami and Jackson Memorial Medical Center, 1611 NW 12 Avenue West Wing 279; LC: R-109, Miami, FL 33136 USA; 3 Cardiovascular Institute, Beth Israel Deaconess Medical Center, 185 Pilgrim Road Palmer 4, Farr Building, Boston, MA 02215 USA; 4 Department of Cardiology, Beth Israel Deaconess Medical Center, 330 Brookline Ave-Shapiro, Boston, MA 02215 USA

**Keywords:** TAVR, Aortic annulus, MDCT, ECG-gating, Multiplanar reconstructions

## Abstract

**Background:**

Transcatheter aortic valve replacement (TAVR) is a novel technique developed in the last decade to treat severe aortic stenosis in patients who are non-surgical candidates because of multiple comorbidities.

**Methods:**

Since the technique is performed using a transvascular approach, pre-procedural assessment of the aortic valve apparatus, ascending aorta and vascular access is of paramount importance for both appropriate patient selection and correct device selection. This assessment is performed by a multi-disciplinary team with radiology being an integral and important part.

**Results:**

Among imaging modalities, there is growing scientific evidence supporting the crucial role of MDCT in the assessment of the aortic valve apparatus, suitability of the iliofemoral or alternative pathway, and determination of appropriate coaxial angles. MDCT also plays an important role in post-procedure imaging in the assessment of valve integrity and position.

**Conclusion:**

This review outlines the principal aspects of TAVR, the multidisciplinary approach and utilisation of different imaging modalities, as well as a step-by-step approach to MDCT acquisition protocols, reconstruction techniques, pre-procedure measurements and post-procedure assessment.

**Teaching Points:**

• *TAVR is a new technique to treat severe aortic stenosis in high-risk and nonsurgical candidates.*

• *MDCT assessment of the aortic annulus is important for appropriate patient and device selection.*

• *Multidisciplinary approach is required for patient selection, procedure planning and performance.*

• *MDCT is required for assessment of the aortic root, iliofemoral or alternative vascular pathway.*

## Introduction

Aortic stenosis is the most common valvular disorder in developed countries, affecting 2–5 % of the population over 75 years old [1, 2]. Once symptoms develop, the mortality increases rapidly to approximately 50 % within 2 years [3, 4]. The standard of care in treatment of patients with symptomatic severe aortic stenosis remains surgical aortic valve replacement (sAVR), which has low peri-operative mortality and improved clinical outcomes [5]. However, high-risk patients often have significant comorbidities such as coronary heart disease, renal insufficiency, lung disease, cerebrovascular disease or frailty that limit their chance of survival [6, 7]. The most commonly used scoring tool to estimate 30-day mortality after cardiac surgery is the Society for Thoracic Surgery Predicted Risk of Mortality score (STS PROM) [8]. For the patient population considered unsuitable or at high-risk for sAVR because of underlying comorbidities, the development of transcatheter aortic valve replacement (TAVR) has resulted in an alternative therapy for symptom relief and extension of life [9].

## Aortic valve apparatus anatomy

**Fig. 1:**
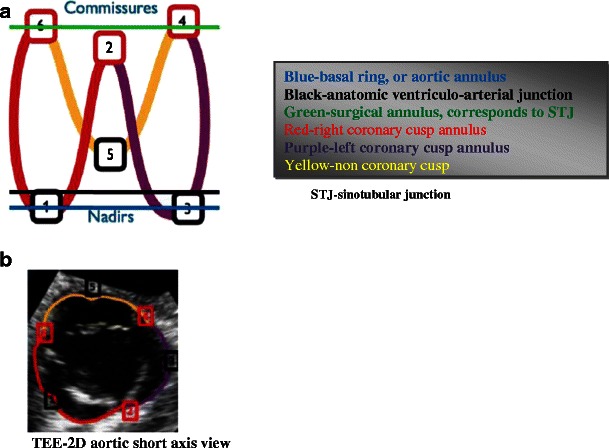
Aortic valve complex: schematic representation and correlation with transesophageal echocardiography (TEE). The aortic valve complex is a formation consisting of the aortic valve annulus, commissures and sinotubular junction (STJ). Three aortic valve cusps are connected proximally to the wall of the left ventricular outflow tract (LVOT) by three anchor points at the nadir (hinge point) of each aortic cusp. Those nadir points, when virtually connected, form an oval-shaped, three-pronged coronet—the “virtual basal ring”

The aortic valve complex consists of the aortic valve annulus, commissures, sinuses of Valsalva (SOV), coronary ostia (CO) and sinotubular junction (STJ). Three aortic valve cusps are connected proximally to the wall of the left ventricular outflow tract (LVOT) [10] by three anchor points at the nadir (hinge point) of each aortic cusp. Those nadir points, when virtually connected, form an oval-shaped, three-pronged coronet—the “virtual basal ring” (Fig. 1). The orientation of this virtual basal ring, or aortic annulus, is double oblique and does not correspond to conventional axial, coronal or sagittal planes of MDCT. This ring is the major target for transcatheter aortic valve prosthesis sizing.

Distally, the three semilunar leaflets are attached to the wall of the aortic sinus at the surgical annulus that corresponds to the sinotubular junction: the junction between the SOV and ascending aorta. Multiple manipulations of the raw imaging data are required to create an image that would exactly correspond to the aortic annulus (virtual basal ring). This virtual basal ring is often not orthogonal to the LVOT, and insertion of the right coronary cusp can often be inferior to the left and non-coronary cusps [11].

## TAVR: review of the device and procedure

**Fig. 2:**
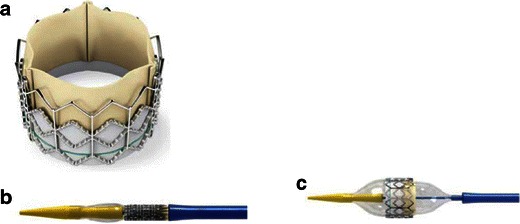
**a** Balloon-expandable Edwards SAPIEN transcatheter heart valve with bovine pericardial leaflets. The first generation frame was made of stainless steel and the newer version Edwards SAPIEN XT THV is made of cobalt chromium. **b** The Edwards Sapien THV crimped on delivery catheter balloon. **c** Fully balloon expanded THV. (Source: Edwards Lifesciences)

**Fig. 3:**
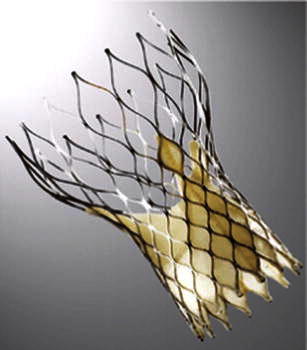
Medtronic CoreValve Transcatheter Heart Valve composed of a self-expanding nitinol stent structure with porcine pericardial valve leaflets. (Source: Medtronic)

**Fig. 4:**
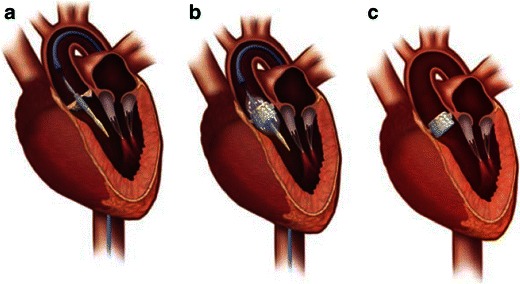
Transfemoral access approach using the Edwards SAPIEN transcatheter heart valve. **a** THV crimped onto a balloon at the tip of the delivery catheter positioned across the native aortic valve. **b** Expanded balloon with THV deployed in position. Angiographically the aim is for a 50:50 deployment with half of the device above and half below the annular plane. **c** The Edwards SAPIEN transcatheter heart valve in correct position in situ. (Source: Edwards Lifesciences)

There are currently two transcatheter heart valves (THVs) available for use in the US: the Edwards SAPIEN valve (Fig. 2) and the Medtronic CoreValve prosthesis (CoreValve) (Fig. 3). Both devices were granted Conformité Européenne (CE) approval in Europe in 2007, and the Edwards SAPIEN valve was approved for commercial use in the US in 2011 following the landmark PARTNER trial [12, 13]. The CoreValve device is currently available in research trials only. The main characteristics of both devices are summarised in Tables 1 and 2, with some specific features outlined below. Both devices are predominately delivered through a transfemoral approach involving either percutaneous or surgical cutdown for arterial access (Fig. 4).

**Table 1:** Comparison of the Edwards SAPIEN XT and Medtronic CoreValve Prostheses

Characteristics	Edwards SAPIEN XT	Medtronic CoreValve
Frame	Cobalt chromium	Nitinol
Leaflets	Bovine pericardial	Porcine pericardial
Expansion	Balloon expandable	Self-expanding
Repositionable	No	Yes
Retrievable	No	Yes
Annular/valvular fixation	Yes	Yes
Ascending aorta fixation	No	Yes
Sheath internal diameter	18-F, 19-F	18-F
Sheath external diameter	7 mm	7 mm
Minimal arterial diameter	6 mm^a^	6 mm
Suitable for		
Dilated ascending aorta	Yes	No
Transapical access	Yes	No
Transaxillary access	Yes, limited experience	Yes, limited experience
Transaortic access	Yes	Yes
Longest published follow-up	>6 years	>4 years
Pacemaker requirement	3 %–8 %	14 %–40 %
FDA approval	SAPIEN transfemoral only	No
Randomised trial results	PARTNER A and B	Results anticipated 2013

^a^Edwards SAPIEN XT 29 mm requires peripheral vessel patent lumen diameter of >7 mm

Modified from [63]

**Table 2:** Optimal aortic annulus, aortic root and peripheral vessel lumen dimensions for different types and sizes of THV

	Aortic annulus diameter, mm	Distance aortic annulus to left main ostium, mm	Ascending aorta diameter, mm	Sinus of Valsalva height/width, mm	Peripheral vessels patent lumen diameter, mm	Valve height, mm
Edwards SAPIEN XT 23 mm	18–22	≥10			>6	14.3
Edwards SAPIEN XT 26 mm	21–25	≥10			>6.5	17.2
Edwards SAPIEN XT 29 mm	24–27	≥10			>7.0	19.1
Medtronic CoreValve 23 mm	18–20		≤40	≥15/≥27	>6	45
Medtronic CoreValve 26 mm	20–23		≤40	≥15/≥27	>6	53
Medtronic CoreValve 29 mm	23–27		≤43	≥15/≥29	>6	55
Medtronic CoreValve 31 mm	26–29		≤43	≥15/≥29	>6	52

*THV* transcatheter heart valves

Modified from [11] and [63]

**Fig. 5:**
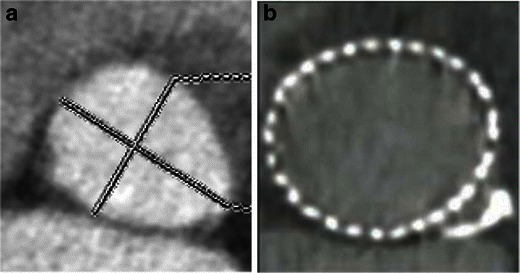
Aortic annulus shape before and after TAVR. Aortic annulus has an oval shape with maximum and minimum diameters (**a**). The minimum diameter usually corresponds to the diameters measured with TTE and TEE. This oval shape will be transformed into a more circular shape after TAVR is performed (**b**)

The Edwards SAPIEN is a ballon-expandable stent. The earlier generation valve is available in 23- and 26-mm valve sizes and is delivered via a 22- or 24-French delivery system, respectively. A newer version, the SAPIEN XT, is available in 23-, 26- and 29-mm sizes using a smaller transfemoral delivery sheath system (18 and 19 French, which requires a minimal iliofemoral luminal diameter of 6 mm). Due to its short stent frame length, the Edwards SAPIEN valve can be deployed in the aortic annulus without any significant limitations in terms of sinus of Valsalva size, sinotubular junction or ascending aortic diameter. The balloon-expandable frame assumes its shape with a single inflation, and the aortic annulus typically conforms to the shape of the device with a more circular geometry (Fig. 5).

Available in 23-, 26-, 29- and 31-mm sizes, the Medtronic CoreValve device (Table 1) is a self-expanding prosthetic valve requiring an 18-French delivery catheter (a minimal iliofemoral luminal diameter of 6 mm). The longer CoreValve device frame requires sufficient ‘outflow’ room to be deployed but the device is available in a wider range of sizes allowing patients with particularly larger annular diameters to be treated. The radial force of the self-expanding frame holds the device in position at the level of the annulus. This same radial force is thought to increase the need for a permanent pacemaker because of compression on conduction tissues, an issue more prevalent with the CoreValve device.

Depending on the specific device, there are several potential additional vascular access routes for implantation [14]:

1. The transapical approach is the second most common access route for TAVR using the Edwards SAPIEN valve (Fig. 6). It essentially affords a direct path to the aortic valve via surgical thoracotomy. While refinements in apical-purse string suturing techniques have reduced bleeding complications, this remains an invasive approach in elderly frail patients.

**Fig. 6:**
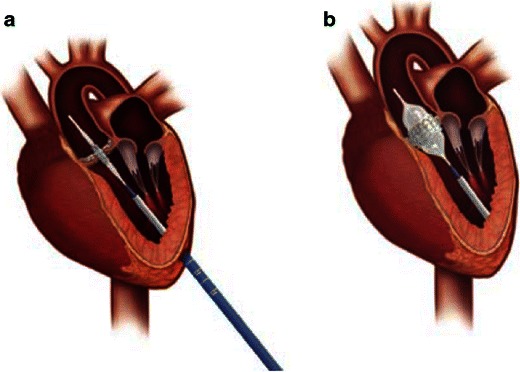
Transapical access using the Edwards SAPIEN THV. **a** Delivery device in situ with crimped device on balloon across the native aortic valve. **a** Left thoractomy is used to gain access to the left ventricular apex and the delivery catheter is advanced with a purse-string suture technique for haemostasis. **b** Expanded balloon with deployed aortic valve prosthesis in correct position. (Source: Edwards Lifesciences)
2. The direct aortic approach was originally developed for use with the CoreValve device as the larger stent frame precluded the transapical route. Although this still requires a partial sternotomy or right anterior thoracotomy, this is arguably less invasive than the incision required for the transapical route and is gaining favour (Fig. 7) [15].

**Fig. 7:**
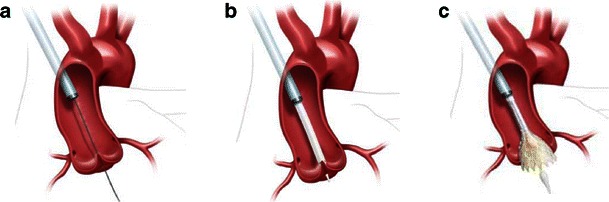
Direct aortic access approach using the Medtronic CoreValve Transcatheter Heart Valve. **a** Arterial puncture and cannulation 7.5–8.0 cm above the level of the aortic annular plane from the right side via a mini-sternotomy. Shown with stiff wire across the native aortic valve and positioned in the left ventricular cavity. **b** Large-bore arterial access sheath in situ with THV delivery catheter crossing the native aortic valve. **c** Partially flared Medtronic CoreValve THV within the native aortic valve. (Source: Medtronic)
3. The subclavian/axillary approach was originally developed for use with the CoreValve device; it can be used for the Edwards SAPIEN prosthesis. With CoreValve device this approach has been losing ground to direct aortic access in recent years. Disruption and dissection are more common with catheter manipulation in the subclavian artery, and caution is required in patients with previous coronary artery bypass grafting in which the left internal mammary artery was used [16, 17]. There are reports describing brachiocephalic access as an alternative approach [18].

**Fig. 8:**
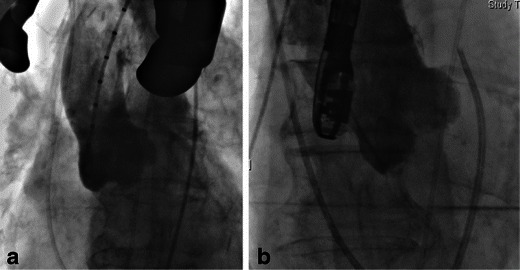
Intraprocedural fluoroscopy. Procedural fluoroscopy demonstrating a pigtail catheter in the non-coronary cusp of the aortic root, with aortography demonstrating the sinus of Valsalva and ascending aorta. The pigtail catheter is used for intermittent aortography during the procedure to aid in correct device positioning and also to determineTransoesophageal haemodynamics and degree of aortic incompetence. **a** Baseline aortography during a direct aortic access procedure (incision ‘spreaders’ shown). Aortography aids in identification of the optimal position for aortic puncture needed to introduce the large-bore arterial access sheath. **b** Baseline aortography with Swan-Ganz catheter in situ in the pulmonary artery from right internal jugular access and transesophageal echocardiography probe

**Fig. 9:**
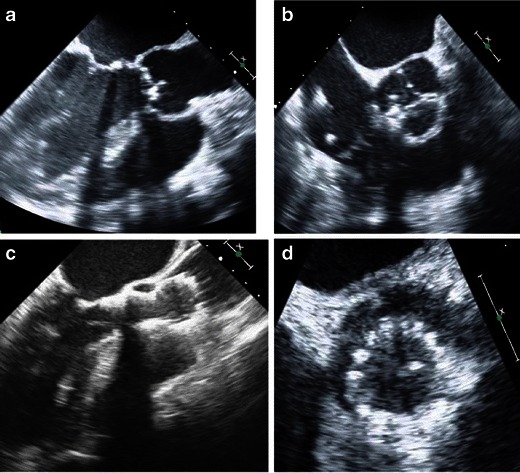
Intraprocedural baseline transesophageal echocardiography and echocardiographic imaging with the CoreValve THV in situ. **a** Long-axis view showing heavily stenosed aortic valve. **b** Short-axis imaging showing a calcified and stenosed aortic valve in systole with poor leaflet opening and small aortic valve opening area. **c** Transesophageal long-axis view showing the CoreValve THV frame with the inflow portion positioned in the left ventricular outflow tract/aortic annulus and the outflow (wider frame) within the aortic root/proximal ascending aorta. **d** Transesophageal short-axis view showing inflow of the device frame in position within the left ventricular outflow tract

TAVR is performed with cardiac surgery back-up and typically general anaesthesia is used. Imaging during the procedure is crucial for accurate device positioning, assessing prosthetic valve function and efficient detection of procedural complications. Positioning of the prosthetic valve is aided by pre-determination of the annular plane orientation using pre-procedure MDCT and rotational angiography with fluoroscopy during the procedure (Fig. 8). Transesophageal echocardiography (TEE) plays an important role in device positioning, final placement and determiningTransoesophageal degree of aortic regurgitation following deployment (Fig. 9). Main procedure risks and complications include vascular access complications, aortic valve regurgitation, transvalvular and paravalvular, stroke, conduction system abnormalities, prosthesis embolisation, mitral valve disruption, haemorrhage, peripheral vascular complications and death [19, 20].

## Patient selection for TAVR suitability

Currently TAVR is reserved for patients with symptomatic severe aortic stenosis in whom pre-existing comorbidities increase the risk of sternotomy and standard valve replacement, and who are considered high or very high surgical risk (Table 3). Aortic annular size appropriate for any of the existing THV devices is an important inclusion criterion. Multiple objective estimates of surgical mortality are applied in the selection process of patients referred for TAVR [21–23]. A logistic EuroSCORE (European System of Cardiac Operative Risk Evaluation) of 20 points or a Society of Thoracic Surgeons Score of 10 points is often used as a risk threshold to aid in decision making [23]. As of 2013, there had been more than 40,000 transcatheter valvular replacements performed worldwide and approximately 7,000 cases performed in the US.

**Table 3:** Inclusion and exclusion criteria for TAVR

Inclusion criteria:
Critical aortic stenosis (with mean aortic valve area of <0.8 cm^2^)
Multiple comorbidities with 1-year mortality rate exceeding 20 %
Poor outcome with medical management
Non-surgical candidates with TAVR representing the only suitable alternative
Native aortic annular size appropriate for currently available THV size criteria
*Exclusion criteria: unsuitable native anatomy*
Lack of appropriate access to implant the device
Sinuses of Valsalva unable to accommodate prosthetic valve
Native aortic annular size inappropriate for currently available THV size criteria

Annular size not corresponding to any of the available THV devices would be a major exclusion criterion, followed by the lack of an appropriate vascular access route; for the CoreValve device, this criterion would be sinus of Valsalva dimensions too small or too large to accommodate the upper frame when positioned (Table 3).

## Pre-TAVR assessment of aortic root anatomy with echocardiography and MDCT

**Fig. 10:**
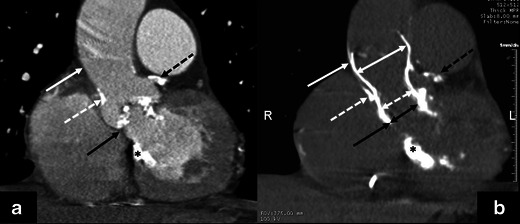
An 81-year-old male following TAVR with a 31-mm Medtronic CoreValve*. Coronal oblique MPR of an enhanced MDCT before TAVR (**a**) and coronal oblique thick MPR of a non-enhanced MDCT following TAVR (**b**). Key anatomic landmarks: Native aortic valve annulus plane (*solid black arrow*), left main coronary artery emanating from the left coronary sinus of Valsalva (dotted black arrow), sinotubular junction (*dotted white arrow*), ascending aorta approximately 4 cm above the native aortic valve annulus plane (*solid white arrow*); Medtronic CoreValve (*double-headed arrows*) and mitral valve annular calcifications (*). Note normal positioning of the Medtronic CoreValve with the upper portion at ascending aorta (*double-headed white solid arrow*), mid portion at the sinuses of Valsalva (*double-headed dotted white arrow*) and lower portion, “inflow” aspect at or just below the native aortic valve annulus plane (*double-headed solid black arrow*)

**Fig. 11:**
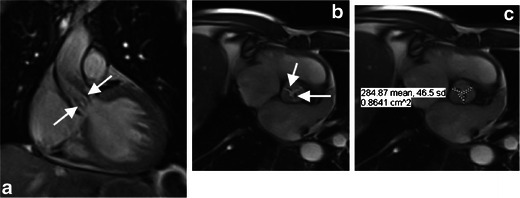
A 66-year-old male with aortic stenosis. **a** Steady-state free precession MR image at peak systole in coronal plane shows hypointense jets in the aortic root (*white arrows*), which result from dephasing of turbulently flowing blood, consistent with haemodynamically significant aortic valve stenosis. **b** Steady-state free precession MR image at peak systole in short axis plane of aortic valve shows a tricuspid aortic valve with valve leaflet thickening because of sclerosis and/or calcifications (*white arrows*). **c** Planimetry in the short axis plane of the aortic valve at peak shows maximal valve opening of 0.86 cm², consistent with severe aortic valve stenosis. MR imaging is particularly useful for TAVR candidates with renal failure since necessary measurements can be obtained without administration of gadolinium

Two- and three-dimensional transthoracic (TTE), transesophageal echocardiography (TEE), ECG-gated MDCT and rarely cardiac magnetic imaging (CMR) (Figs. 10 and 11) can be used for aortic valve apparatus assessment, with each imaging technique providing complementary information for optimal valve prosthesis sizing and THV selection. TTE, TEE and MDCT are currently the primary modalities for aortic root anatomy assessment.

### Echocardiography assessment

Echocardiography is used to confirm severe aortic stenosis (aortic valve area of <0.8 cm^2^, peak velocity across valve >4 m/s and mean gradient >40 mmHg). It is also used to assess the degree of aortic incompetence and other valvular diseases, left ventricular systolic ejection fraction and diastolic dysfunction, as well as to estimate right-sided and pulmonary pressures. For TTE, the para-sternal long-axis view is used, and for TEE, the mid-esophageal long-axis view is used. The annulus is measured during early systole, with the valve leaflets open, from the hinge point of the right coronary leaflet to the hinge point of the non-coronary leaflet. THree-dimensional TEE may provide more accurate assessments of the aortic annulus compared to 2D-TEE, which may impact prosthesis size selection, although more research in this area is needed [24, 25]. Despite promising results and crucial information, 3D TEE is not yet a standard practice in pre-TAVR aortic annulus measurement assessment. TEE allows for real-time imaging during the TAVR procedure while assisting in device placement and positioning as well as assessing the degree of aortic regurgitation [12]. Other complications such as pericardial tamponade, severe mitral regurgitation, aortic dissection, LV damage and embolisation of the implanted valve can also be diagnosed intra-procedurally with TEE. [12].

### MDCT assessment

The Society of Cardiac Computed Tomography (SCCT) expert consensus document on MDCT imaging before TAVR suggests using at least a 64-detector scanner, with an obvious advantage of 128, 256 and 320 slice scanners with their shortened acquisition time and decreased contrast volume [11, 26] and high-pitch spiral dual-source CT angiography protocol [27]. Imaging should be performed in the supine position and during suspended respiration [11]. Since precision in the range of 1 mm is desirable, spatial resolution must be high with an acquisition protocol that obtains a reconstructed slice width of ≤1.0 mm throughout the entire imaging volume, particularly of the aortic valve, aortic root and ilio-femoral arteries.

## Aortic annulus apparatus imaging

For precise aortic valve annular sizing and root evaluation, ECG-gated CT angiograms of the ascending aorta and heart are obtained with either prospective or retrospective ECG triggering. Aortic valve and root assessment during systole has been shown to be preferable to during diastole because of the larger annular size noted in systole as well as dynamic changes [28, 29]. Thus, with both prospective and retrospective gating, the data should be acquired during systole (usually 20–50 % phase of the cardiac cycle) with no radiation during the rest of the cycle in prospective ECG triggering and very aggressive dose modulation with retrospective gating, allowing substantial dose savings. No routine administration of β-blockers is used for scanning purposes because of concerns with underlying severe aortic stenosis. A high incidence of arrhythmia in TAVR candidates precludes routine use of prospective gating on a routine basis. As a result, the estimated radiation dose may be relatively high, but acceptable given the advanced age of the vast majority of TAVR candidates and the amount of information acquired. For younger patients, with stable and low heart rates, prospective axial acquisition during the systolic phase is suggested [26]. Tube potential of 100 kV is suggested for patients weighing less than 90 kg or with a body mass index (BMI) less than 30, whereas a tube potential of 120 kV is usually indicated for patients weighing more than 90 kg (BMI >30). The lowest setting possible should be selected in keeping with acceptable image noise [30].

**Image acquisition** triggering can be obtained automatically or by using a bolus injection technique for determining the transit time. The region of interest (ROI) can be placed on the proximal descending aorta using a pre-selected HU threshold. Arterial enhancement in studies performed with a 64-slice scanner demonstrates more consistency with the bolus injection technique, but requires higher contrast volume (typically an additional 20 ml of contrast medium). The vast majority of medical centres imaging TAVR candidates follow one of the two two-step protocols (Table 4). Reduction of contrast volume can be achieved by using lower flow rates such as 3 ml/s or less [31]. Dual-energy techniques allow adequate enhancement with lower intravenous contrast volumes [32]. In extreme cases of renal insufficiency, direct aortic injection with extremely low volumes of contrast (,20 ml) can be used [33, 34].

**Table 4:** Suggested IV contrast injection regiments

64-Detector scanner	320-Detector scanner
Gated cardiac CTA	Gated CTA chest
Bolus injection, 20 ml IV contrast	Automatic triggering
ROI: proximal descending aorta	ROI: proximal descending aorta
Peak enhancement selected	Threshold of +200 HU used
Main injection: 4 ml/s, 70–80 ml	Rate: 4 ml/s, 120–140 ml
Non-gated CTA abdomen, pelvis	Non-gated CTA abdomen, pelvis
Additional 50 ml is injected	Done immediately after chest CTA
Injection timing based on bolus results	No additional IV contrast required
Total:	Total:
130–150 ml	120–140 ml

*ROI* Region of interest

**Fig. 12:**
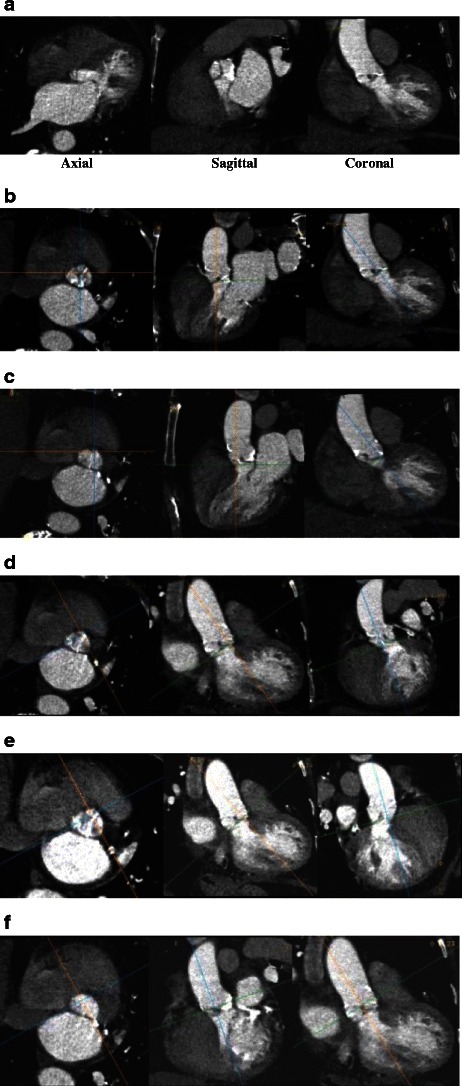
Sequence of images describing the step-by-step process of creating a plane that precisely corresponds to the aortic annulus [17, 24]. **a** Thin-sliced axial reconstructions (0.625 mm for GE and 0.5 mm for Toshiba) are used to create standard coronal, sagittal and transverse plane reconstructions. **b** Coronal plane reference line is rotated to bring the former axial plane as close as possible to the plane of the valve. **c** The reference line in the coronal image that controls the former axial plane is moved up and down to identify the lowest insertion point of the right coronary cusp, and the former axial plane is positioned exactly at the level of the right cusp insertion point. **d** The reference line in the formerly axial plane is rotated such that the line that controls the former sagittal plane crosses the lowest insertion point of the non-coronary cusp (located approximately at the 8 o’clock position, *). **e** Manipulation with the former sagittal plane, currently showing the lowest insertion of the right and non-coronary cusp. On this plane, the reference line of the former axial plane should be rotated until it crosses the two insertion points. **f** On the former coronal plane the reference line of the former axial plane is rotated until the lowest point of the left coronary cusp appears on the formerly axial plane window. At this point the formerly axial plane is now rotated to represent the correct orientation and level of the aortic valvular plane. This image should be used to provide all the precise pertinent measurements

**Fig. 13:**
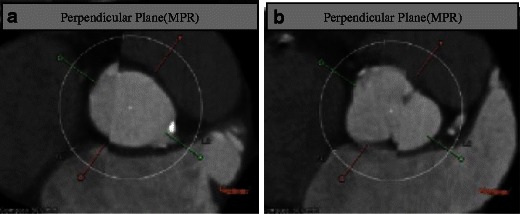
Suboptimal imaging of the aortic annulus in a patient with irregular high heart rate. An 85-year-old female with rapid atrial fibrillation. Due to the irregular heart rate, substantial image registration artefacts severely degraded image quality of CT raw data as well as subsequent post-processing images. Thus, all the required measurements—(**a**) aortic annulus and (**b**) width of the SOV, cannot be reliably assessed

Image reconstruction of the aortic valve apparatus requires a complex reconstruction technique based purely on aortic valve anatomy. No single image reconstruction protocol exists, but similarities between clinical centres and approaches exist. Currently the most widely used method for reconstruction of aortic valve annulus is based on sequential double-oblique reconstructions that ultimately provide the image corresponding to the aortic valve annulus. The post-processing approach used for assessment of the aortic root and ascending aorta has been described in detail by Achenbach et al. and Leipsic et al. (Fig. 12) [11, 26]. As with all other types of CT post-processing, the quality and precision of reconstructed images depend on the quality of the raw CT data. The presence of a high heart rate, arrhythmia and severe valve calcifications can substantially degrade image quality (Fig. 13). If these limitations present, they should be addressed in the report, and a decision should be made on the necessity of repeated imaging or acquiring the data with alternative imaging modalities.

**Fig. 14:**
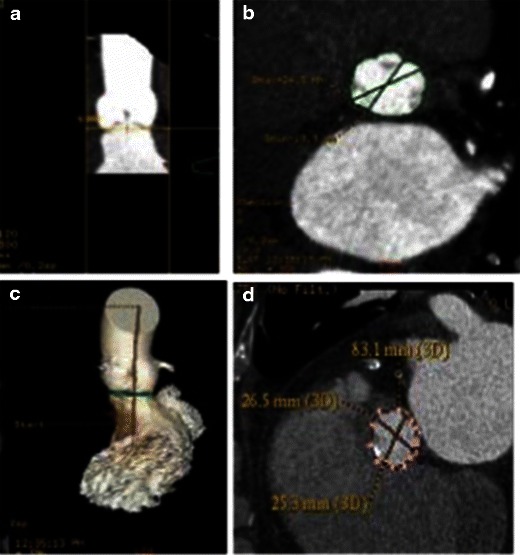
Assessment of aortic annular plane. **a** Aortoventricular centreline is created manually. **b** Based on a centreline, double-oblique MPR images are created at the pre-selected level of the basal annular plane (AA) with all the AA measurements obtained at this level. **c** The 3D volume-rendering reformats of the aortovalvular complex are generated based on the same centreline. **d** Manual measurements of maximum and minimum aortic annulus diameter and perimeter

**Fig. 15:**
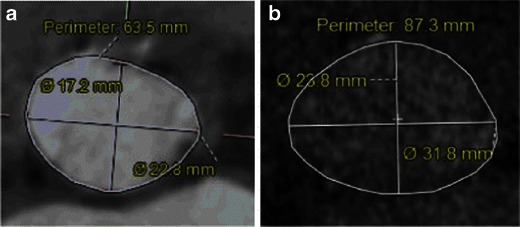
Examples of different sizes of aortic annulus corresponding to different sizes of Medtronic CoreValve prostheses. The measurements of the aortic annulus shown above would be appropriate for (**a**) Medtronic CoreValve 23 mm, (**b**) Medtronic CoreValve 31 mm

Measurements of the aortic valve as well as qualitative assessment should be done based on properly reconstructed images (see above). Three main measurements have been suggested as being most informative regarding aortic annular sizing and THV selection (Table 5) (Fig. 14): (1) Aortic annulus diameters: long and short diameters are measured, and the mean diameter is calculated by averaging short and long diameters [35]. (2) The circumference (perimeter) of the aortic annulus can be either calculated under the assumption that the area corresponds to full circularity or measured manually (Fig. 15). (3) Aortic annulus area can be calculated based on the mean diameter or measured manually using the planimetry technique. Aortic annulus dimensions should be measured in systole, similar to echocardiography, since both the area and mean diameters are larger in systole [36]. No substantial difference in circumference was found across the cardiac cycle [26, 37].

**Table 5:** Aortic valve apparatus/ascending aorta measurements pertinent for TAVR

Aortic annulus (AA) (virtual basal ring)
• AA maximal diameter
• AA perpendicular minimal diameter
• AA average diameter
• AA cross-sectional area (CSA)
• AA circumference
Aortic valve
• Cuspidity
• Comissure calcifications
• Aortic annulus calcifications
• Severely calcified cusp that might compromise coronary the artery ostia: yes/no
Ascending aorta
• Width at 40 mm from the annulus
• Position relative to the sternum
Sinuses of Valsalva
• Maximum diameter
• Height
• Sinotubular junction maximum diameter
• Distance from the aortic annular plane to the coronary artery ostia

Additional aortic valve apparatus measurements pertinent for TAVR dimensions include the aortic valve cusps, aortic valve commissures, sinuses of Valsalva, coronary ostia and sinotubular junction. The parameters important for pre-TAVR assessment are summarised in Table 6. The most important factors are discussed below.

**Table 6:** Crucial factors for assessing the aortic and peripheral vascular access

Minimal patent arterial luminal short axis diameters
• Aorta
• Common iliac arteries
• External iliac arteries
• Common femoral arteries
• Subclavian arteries
• Innominate artery
Arterial tortuosity
• Mild, moderate, severe
• Kinking (tortuosity >90º)
• Obstruction/thrombosis
Arterial calcification
Extent
• Mild, moderate, severe
Pattern
• Circumferential
• Horseshoe
• At bifurcations
• Arterial dissection
• Yes/no
Arterial complex atheromas
• Yes/no

**Fig. 16:**
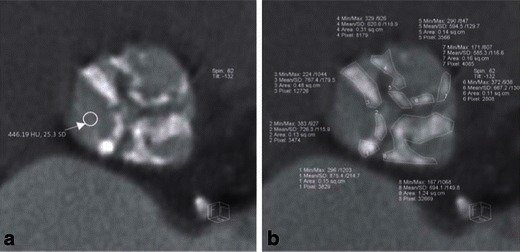
Quantitative volumetric assessment of the native aortic valve apparatus calcification burden based on pre-TAVR ECG-gated cardiac MDCT. This technique requires creation of a series of 3-mm-thick contiguous double-oblique CT images in the short-axis plane of the native aortic valve apparatus that will include the whole volume of the TAVR device landing zone, defined as the area of the aortic valve annulus, valvular cusps and LVOT (up to the junction point of the anterior mitral leaflet) (**a**). At each reformatted image using the planimetry technique, regions of interest (ROI) are drawn around all perceived areas of calcification (**b**). Note that the mean density of all drawn ROIs is significantly higher by more than 2 SD than the mean blood pool density as measured in (**a**), indicating true calcifications. These regions of interest at all levels are added together and multiplied by the slice thickness (3 mm) to derive a quantitative total volumetric calcium score in mm^3^ representing the calcification burden of the entire native aortic valve apparatus

**Fig. 17:**
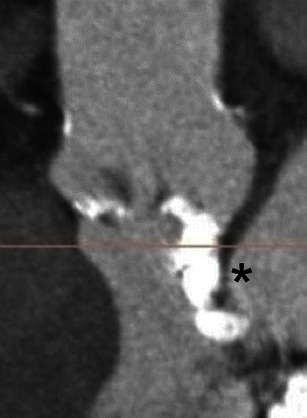
Aortomitral continuity calcification. Calcification of the aortomitral continuity (purple asterisk) that extends to the mitral valve may also increase the risk of annular rupture with post-deployment balloon dilatation, as demonstrated on this double-oblique reconstruction of the aortic annulus

Aortic valve calcifications (AVCs) are either diffusely or focally located on the aortic surface of thickened aortic valve cusps. If excessive, these calcifications may prevent appropriate alignment of the prosthesis, leading to gaps between the prosthetic valve and the aortic annulus and subsequent paravalvular aortic regurgitation (leak), as well as increased risk of annular rupture, device dislodgement or obstruction of coronary ostia due to opposition of calcified aortic cusp [34, 38–42] (Fig. 16). Calcification of the aortomitral continuity may increase the risk of annular rupture with balloon dilatation (Fig. 17). AVC can be assessed in the cross-sectional view of the sinus of Valsalva (SOV) either qualitatively (mild, moderate, severe) or quantitatively using Agatston units (Agatston Score), the calcified volume score (in mm^3^) or mass score (in mg of CAHA), similar to the assessment of coronary calcifications [38, 43]. Agatston scores exceeding 3,000 are correlated with an increased incidence of paravalvular regurgitation after TAVR [44]. Semiquantitative scores assess the number of affected cusps, homogeneous versus more focal distribution of calcium and involvement of coronary versus non-coronary cusps. Calcium burden is quantified at the aortic annulus and at the SOV at both its inferior portion and superior aspect. Post-deployment balloon dilatation is required more frequently in patients with severe valvular calcification [45].

**Fig. 18:**
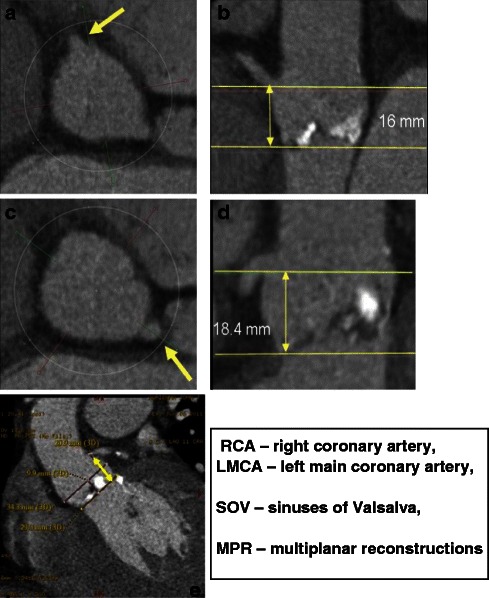
Distance of the coronary ostia to the aortic valve plane–coronary artery height. The height of the right coronary ostium is determined by identifying the origin of the RCA from the transverse MPR parallel to the aortic annulus plane (**a**) and then measuring the height of the coronary artery above the basal annular plane (**b**). In a similar fashion, the origin of the LMCA is identified from the transverse MPR (**c**) and then measuring the height of the LMCA above the basal annular plane (**d**). In this case, the LMCA origin is above that of the RCA. (**e**) Relationship between the height of the LMCA origin and the maximum width of the SOV and aortic annulus plane

Distance of the coronary ostia to the aortic annulus plane: Coronary arteries generally arise within the SOV, below the level of the sinotubular junction (STJ). The right coronary artery generally lies higher than the left coronary artery, with average distance between the plane of the aortic annulus and coronary artery ostium (CAO) of 15.5 mm on the left and 17.3 mm on the right [46]. Degenerative aortic stenosis may result in longitudinal remodeling of the SOV with decreased distance from the aortic valve annulus to the CAO (Fig. 18). The combination of a relatively low-lying coronary artery ostium and a large native aortic valve leaflet can therefore obstruct the flow into the coronary arteries during device deployment. The height of the CAO is determined by identifying the origin of the coronary artery from the cross-section MPR and then measuring the vertical distance between the inferior edge of the CAO and the aortic annular plane. If coronary ostia height is more than 10–14 mm in patients with planned THV implantation, the chance of obstruction is low [11].

**Fig. 19:**
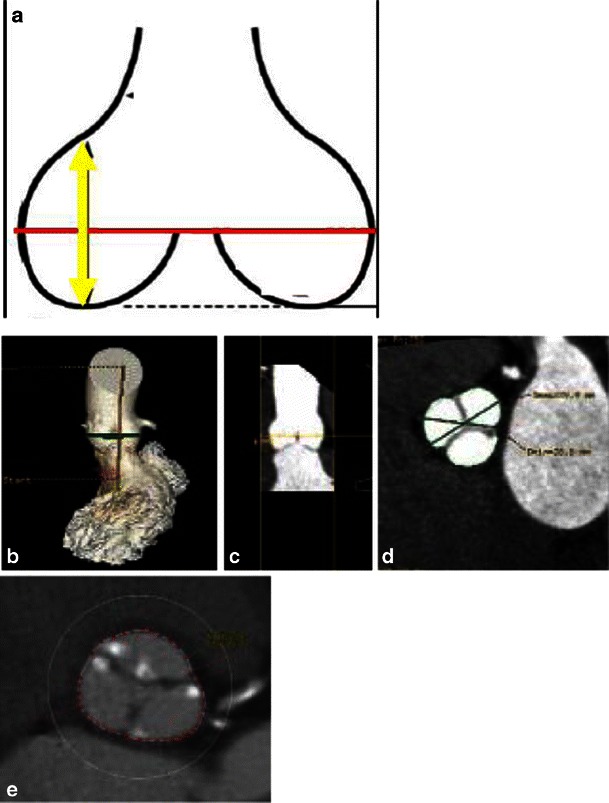
Measurements of sinus of Valsalva height, width and perimeter. The widths (**a**, *red line*) and maximal height (**a**, *yellow arrow*) of the SOV are important parameters for coronary perfusion after TAVR as they determine whether the valvular prosthesis will be accommodated within the SOV without causing coronary occlusion from displacement of the native valve leaflets. First, to measure the SOV parameters, 3D volume-rendering reconstruction is created, with the area of interested selected at the maximum diameter of SOV (**b**, *green line*). Then, based on curved multiplanar reconstruction (**c**), a true perpendicular image is created (**d**) where the maximum diameter is measured. The perimeter of the SOV can also be assessed on the same image (**e**)

The width and maximal height of the SOV are important parameters for coronary perfusion with THV, determining whether the THV will be accommodated within the SOV without causing coronary occlusion from displacement of the native aortic valve leaflets [47] (Fig. 19). As for the rest of the aortic valve apparatus, measurements of the aortic sinus diameter and height should be assessed on a double-oblique projection. The SOV width assessment is particularly important for the CoreValve device with a minimum 27 mm required for the 26-mm prosthesis and 29 mm for the 29- and 31-mm prostheses; the minimum SOV height is 15 mm (Table 6) [26].

The diameter of the ascending aorta: For CoreValue devices, orientation of the direction of the device flow occurs when the frame contacts the inner and outer curvature of the aorta. Ascending aortic dilatation of more than 43 mm (when measured 4 cm above the basal aortic annulus plane) precludes the use of the 29- and 31-mm CoreValve device. Dilatation of more than 40 mm precludes the use of the 23- and 26-mm CoreValve device. Size is of less concern for valves that are short in length and confined to the aortic annulus and sinus of Valsalva such as the Edwards SAPIEN valve [11].

**Fig. 20:**
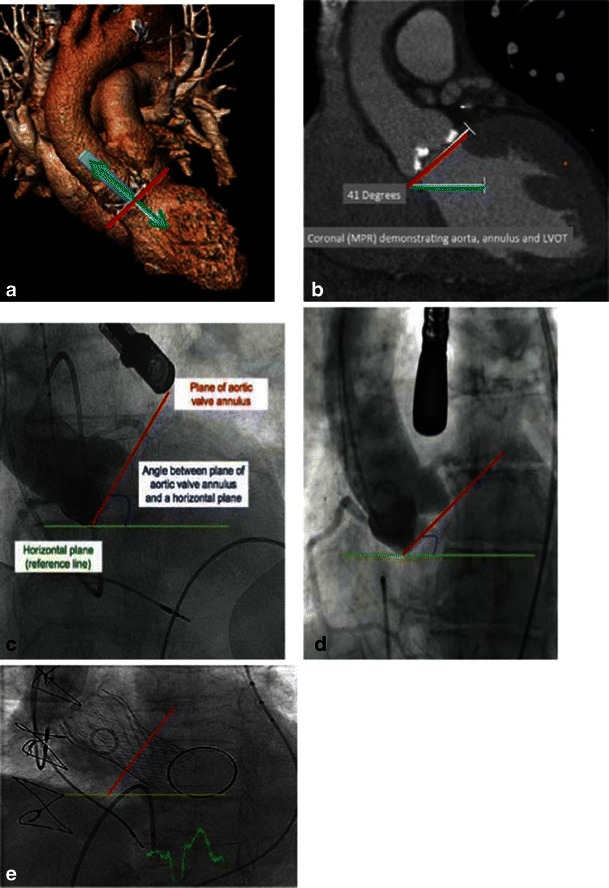
**A-E** Aortoventricular angle assessment. **a** The aortoventricular angle is best determined from the coronal view by determining the angle of the horizontal plane (*blue*) at the level of the ventricle and aortic annulus angulation (*red*). Green: aortic longitudinal access. **b** The aortoventricular angle is determined by the angle of the horizontal plane and the angle of the aortic annulus. In this case, the aortoventricular angle is 41º. (**c, d**) The AV angle can also be established during angiography, although it may be time consuming and disruptive during the actual procedure. **e** The angle can also be seen after deployment of the TAVR catheter (this patient had remote replacement of the mitral valve)

Additional features that might interfere with successful implantation: The left ventricular outflow tract (LVOT) is the area between the basal annulus and the anatomic aortoventricular junction. The atrioventricular (AV) node is located adjacent to the membranous septum of the LVOT below the aortic valve. Deep positioning of the THV into the LVOT may cause injury to the interventricular septal and artrioventricular conduction system. The degree of LV upper septal hypertrophy protruding into the LVOT can hinder accurate placement of the valve and present a significant risk of THV repositioning. The aortoventricular angle is the angle between the proximal aorta, aortic annulus and LVOT (Fig. 20). This angle is determined by the angle of the horizontal plane and the angle of the aortic annulus [26]. The aortoventricular angle is an important consideration when using THVs that are long in length and require deployment perpendicular to the native annular plane. Each type of THV has a maximum aortoventricular angle for successful valve deployment, typically 70º for the iliofemoral approach and 30º for the subclavian approach, due to angulation from the right subclavian into the ascending aorta.

## Peripheral vascular pathway, aorta, chest wall and left heart imaging

Imaging of peripheral access can be obtained with non-gated spiral acquisition to minimise radiation. Peripheral access includes the iliofemoral, transsubclavian, transapical and direct aortic access routes. Transapical and direct aortic access (ascending aorta) routes are assessed with the ECG-gated portion of the MDCT. The common femoral artery should be included in the field of view when assessing femoral access. Assessment of the axillary and subclavian arteries should be obtained with the patient’s arms placed along his/her body to exclude pseudo narrowing of the imaged vasculature.

**Fig. 21:**
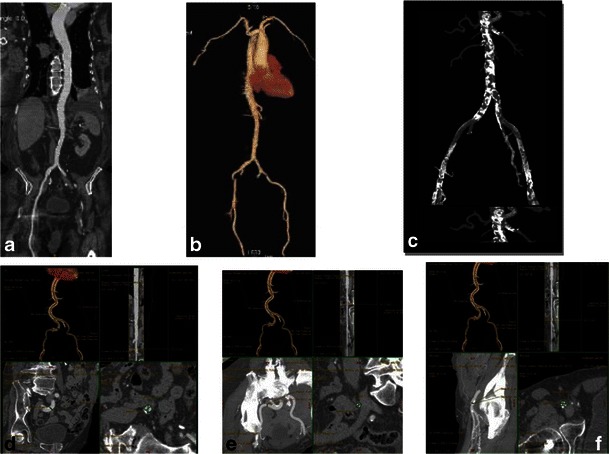
Standard reconstructions for assessment of peripheral vascular pathway. The approach includes first creation of a curved reformat of each artery, including the aorta (**a**), as well as 3D volume-rendered images (**b**). Those reconstructions provide landmarks to display the sites of subsequent measurements of luminal diameter and 3D volume-rendering maximum intensity projection (MIP) reformats (**c**) demonstrate the severity of aortic and ileo-femoral calcifications. Curved multiplanar reformats, obtained with a centreline approach, serve as the source for multiple luminal measurements made in a plane orthogonal to the vessel. **d, e, f** show the process of assessing orthogonal axial planes with measurements of the smallest diameters at each vessel level [common iliac artery (**d**), external iliac artery (**e**), common femoral artery (**f**)]

MDCT findings crucial in the peripheral vascular pathway assessment are summarised in Tables 1, 2 and 6. MDCT selection of patients with appropriate peripheral access has substantially reduced the risk of vascular complications from 30.1 % [12] to 8 % [48]. A sheath-to-femoral artery ratio ≥1.05 has been shown to be associated with increased vascular complications and 30-day mortality [36, 48]. A standardised approach for peripheral access has been developed, adding significant reduction in vascular injury rates [48]. Accepted practice is to evaluate peripheral access with several reconstruction techniques: curved multiplanar reconstructions (MPR), 3D volume-rendered images and, if necessary, maximum intensity projection (MIP) images (Fig. 21). These multiple measurements are taken along the common iliac, external iliac, common femoral, innominate, subclavian and proximal axillary arteries bilaterally.

**Fig. 22:**
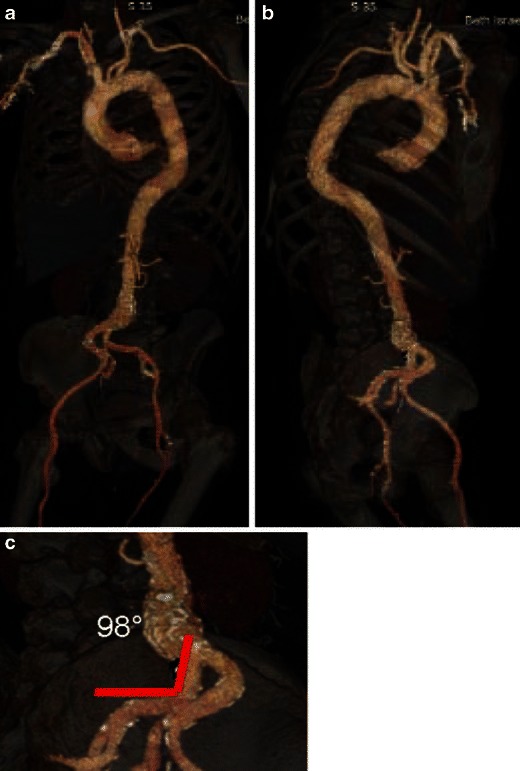
Iliac artery angulation; 3D volume-rendered images of the aorta, subclavian and iliac vessels with skeletal landmarks. Reconstruction provides landmarks to display the sites of subsequent measurements of luminal diameter (**a, b**). The degree of iliac artery ***angulation*** can be measured easily on this type of reconstruction (**c**)

**Fig. 23:**
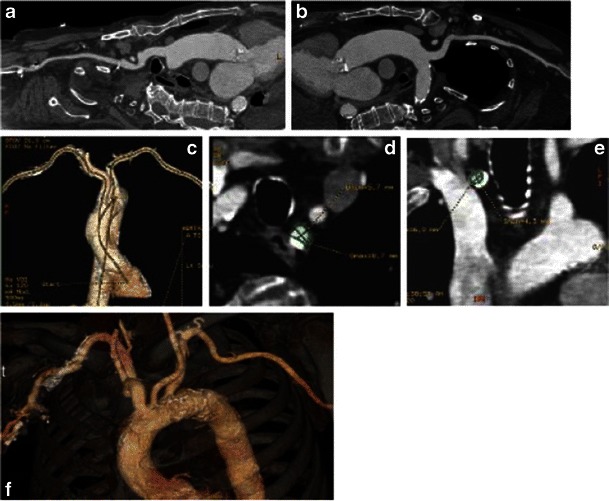
**a-f** Assessment of subclavian arteries. Assessment of subclavian arteries is done in a similar fashion to iliofemoral assessment: centreline approach to both the right (**a**) and left (**b**) subclavian arteries serves as the source for luminal measurements, obtained with 3D VR (**c**), and orthogonal to the vessel (**d, e**) reconstructions with similar parameters assessed, with 3D VR (**f**) reconstructions used to assess the tortuosity and plaque/calcification burden

**Fig. 24:**
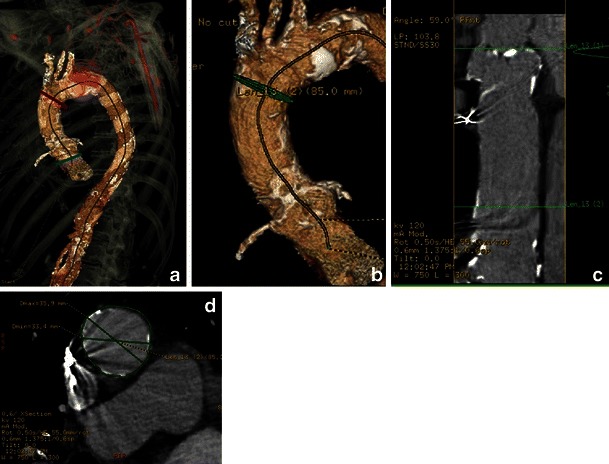
Planning of the direct aortic approach. Assessment of entire ascending aorta is crucial to exclude the presence of thoracic aneurysm or anterior aortic wall calcifications that might potentially interfere with direct puncture of the aorta. Typically, the aorta is punctured within the most proximal 8.5 cm. **a** The 3D VR reconstructions of the ascending aorta for assessment of the diameter and presence of anterior aortic wall calcifications. Bone structures provide landmarks for future thoracotomy. Blue line, aortic valve plane; red line, 8.5 cm above the plane of the aortic valve, showing the area of potential direct aortic access. **b** Magnified view of 3D VR of the ascending aorta with the 8.5-cm landmark, (**c**) curvilinear reconstruction of the ascending aorta with (**d**) automatically created perpendicular to (**c**) the aortic plane with aortic diameter measured at this level

Vessel tortuosity in the iliofemoral or brachiocephalic/subclavian arteries can be optimally assessed using 3D reconstructions, with multiple oblique views seen along the 360° circle in addition to the anterior-posterior view. Precise angle measurements are obtained using PACS or workstation angle measurement tools. Note that vessel angulation of 90° or more may be a contraindication for large-bore catheter insertion (Fig. 22). The burden and pattern of vascular calcifications and presence of circumferential or horseshoe calcification can be better assessed on curved multiplanar and maximum intensity projection (MIP) reconstructions. The combination of relatively small vessel caliber or stenotic segments with these types of vascular calcifications is a relative contraindication for a transfemoral approach. However, a relatively straight segment with no substantial calcification or atheroma can be cannulated even if marginally smaller in diameter than the intended sheath [49]. The presence of dissection or a complex atheroma is assessed on both axial and curved reconstruction images, with such findings again considered contraindications for a transfemoral approach. If the iliofemoral approach is not an option for a given patient, transapical, direct aortic, direct brachiocephalic or subclavian artery approaches should be assessed (Figs. 23 and 24).

**Fig. 25:**
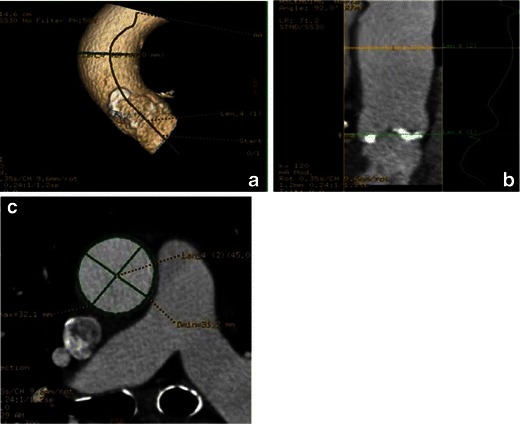
A 76-year-old female with severe aortic stenosis assessed for potential implantation of a Medtronic CoreValve. Aortic diameter 4 cm above the plane of the aortic annulus is crucial: diameter >4.3 cm may prevent even the largest device from successfully anchoring. The 3D volume-rendered images provide landmarks to display the sites of subsequent measurements of the ascending aortic luminal diameter (**a**). This area is carefully measured using a centreline approach (**b**) with subsequent luminal measurements made in a plane orthogonal to the vessel (**c**)

For all TAVR approaches, assessment of the entire aorta is done routinely as part of the pre-procedure protocol using curved MPR with subsequent creation of true perpendicular axial images for precise patent lumen diameter assessment. For the transfemoral approach, the aorta is assessed to exclude the presence of an abdominal aneurysm, elongation with kinking of the aorta, dissection or large thrombi protruding into the lumen and/or complex atheromas, as these are contraindications for transfemoral approach [11]. For the direct aortic approach, assessment of the ascending aorta for the presence and pattern of calcification is of paramount importance, as calcium may interfere with direct puncture of the aorta. In these cases, the ascending aorta from the aortic annulus plane to the origin of the brachiocephalic artery is assessed for the presence of aneurysmatic dilatation or circumferential/anterior calcifications (Fig. 25). The relationship between the aorta and chest is important for planning thoracic chest wall incisions. It is important to assess the retrosternal area for the presence of masses particularly in post-CABG patients to avoid bypass graft injury. After median sternotomy or a right parasternal incision has been performed for the direct aortic approach, the aorta is punctured either through its anterior or right lateral aspects (accordingly), usually within the most proximal 8.5 cm, to allow sufficient room for CoreValve device expansion. MDCT reconstructions routinely include 3D VR reconstructions with osseous structures providing anatomic landmarks for future thoracotomy. For **transapical access**, assessment of the entire aorta is less crucial, unless a change in the approach is anticipated during the procedure. The crucial data are the position of the left ventricle (LV) apex relative to the chest wall and alignment of the LV axis with the LV outflow tract. Also, left atrial (LA) and/or LV thrombi can be a source of embolic complications that will need to be identified and reported. As with the direct aortic approach, chest deformities and mediastinal lesions are important and should be reported and reviewed with the team prior to the procedure.

## Comparative analysis of echocardiography vs. MDCT for aortic valve apparatus assessment

**Fig. 26:**
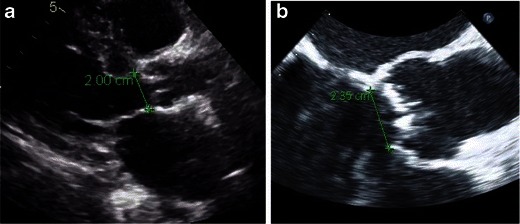
Aortic annular measurement by (**a**) transthoracic echocardiograpy: parasternal long-axis view with measurement shown. **b** Transesophageal echocardiography (TEE): long-axis view shown with annular measurement. Transthoracic echocardiography (TTE) typically underestimates the aortic annulus by approximately 1.0–1.5 mm when compared to TEE or MDCT

**Fig. 27:**
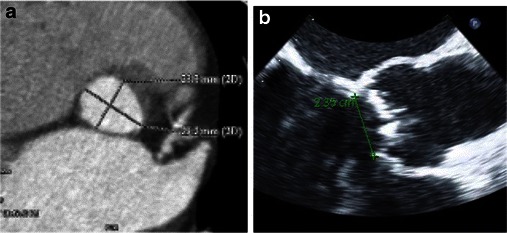
Correlation between MDCT and TEE aortic basal ring measurements. Elliptical shape of the aortic annulus is appreciated on MDCT reconstructions (**a**) with the maximum diameter of the valve corresponding to coronal plane measurement obtained by TEE (**b**). (Note that in this figure the images belong to two different patients)

**Fig. 28:**
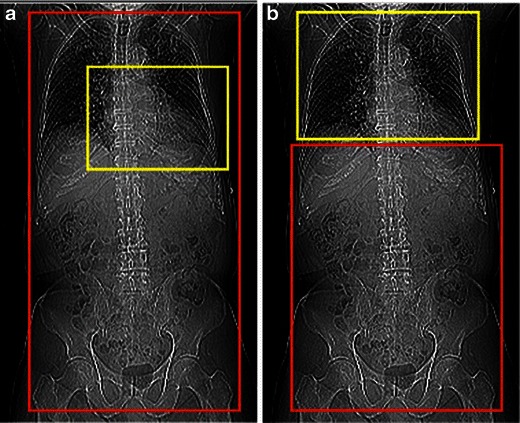
Two types of protocols used based on MDCT scanner parameters: Two scout views corresponding to a 64-slice scanner (**a**) and 320- slice scanner (**b**) demonstrate the approach to data acquisition. First, the gated (yellow rectangle) part of the exam is obtained, including only cardiac CTA with the 64-slice scanner and gated CTA of the chest with the 320-slice scanner. The second step (*red rectangle*) corresponds to CTA chest, abdomen and pelvis in the first scenario (**a**) and CT abdomen and pelvis in the second (**b**). Note that in both cases eventually the entire torso is imaged

Historically, patient eligibility for transcatheter valve therapy and sizing of the prosthesis were largely based upon aortic annulus measurements on 2D echocardiography (transthoracic or transesophageal) and occasionally on angiography. The major disadvantage of these techniques [50–52] is the underestimation of the oval and not circular shape of the aortic annulus (Figs. 26 and 27) [20]. Direct comparison of TEE and MDCT aortic annulus measurements is problematic because of different parameter measures by each of the modalities. Aortic annulus diameter on coronal view by MDCT has been shown to be significantly larger than that obtained on sagittal view by MDCT, TTE or TEE [53]. Long-axis measurement of the aortic annulus by TTE and TEE approximates the minor axis of the elliptically shaped annulus as measured by MDCT (Fig. 28). Thus, consistently larger measurements obtained with MDCT as compared with 2D echocardiography most likely reflect the measurements of the largest of the two diameters of this oval-shaped annulus as opposed to the smaller one measured by echocardiography [54–56]. The 2D echocardiography usually underestimates the aortic annulus average diameter by 1 ± 1.7 mm [26, 52]. As a result of the eccentric geometry of the oval aortic annulus, correlative studies have shown a systematic underestimation of annular sizing by 2D echocardiography alone [57].

MDCT can provide better estimates of both the long- and short-axis diameter of the aortic annulus, surface area and perimeter measurements. Extensive work has been done to establish the role of MDCT in aortic annular sizing with Edward Sapiens transcatheter heart valves (THV) [11, 26, 46]. While exaggerated oversizing of the THV and excessive calcification of the native aortic valve can result in aortic annular rupture, some THV oversizing is required for both types of THV to prevent PAR. For the CoreValve device the recommended device/annulus oversizing is 15 % of the aortic basal ring perimeter. For the Edwards Sapiens valve, the recommended device/annulus oversizing is 15–25 % of the area and 7–12 % of the mean diameter. This degree of oversizing of the THV appears to provide the best risk-benefit ratio in terms of PAR reduction and conduction disorders [57, 58]. Undersizing of the THV can lead to increased PAR and greater likelihood of valve ‘pop-out’ or migration [10, 59].

## Post-implantation imaging

Post-implantation imaging can be divided into immediately post-procedure and long-term follow-up. For the immediate assessment of valve position and haemodynamic status, including the gradients and effective valve area, the modality of choice is TEE, which can be done in the hybrid procedure room. Paravalvular and transvalvular regurgitation can also be estimated in real time, allowing appropriate steps to be taken to minimise complications and optimise device positioning [16, 54].

For long-term follow-up of TAVR ECG-gated MDCT is useful in diagnosing prosthesis misplacement by careful inspection of the exact positioning of the device in relation to the aortic annulus plane [55].

Significantly low implantation may result in severe paravalvular regurgitation, residual aortic valve stenosis, mitral valve insufficiency, conduction abnormalities and, in extreme cases, device drop into the left ventricular cavity. Considerably high implantation may result in paravalvular regurgitation, coronary flow obstruction and device embolisation into the thoracic aorta [10, 60].

**Fig. 29:**
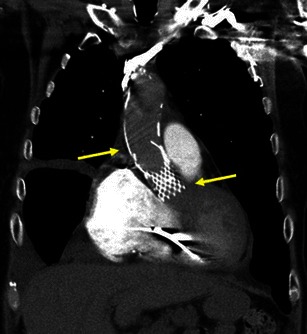
MDCT of the chest following the insertion of the Medtronic CoreValve. The Medtronic CoreValve is normally positioned with its upper portion at the ascending aorta (*upper yellow arrow*), mid portion at the sinuses of Valsalva and lower portion, the “inflow” aspect, at or just below the native aortic valve annulus plane (*lower yellow arrow*)

**Fig. 30:**
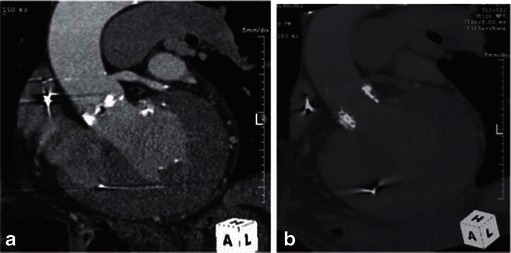
A 58-year-old male following TAVR with a 26-mm Edwards SAPIEN valve and severe paravalvular regurgitation. Coronal oblique MPR of an enhanced MDCT before TAVR (**a**) and coronal oblique thick MPR of an enhanced MDCT following TAVR (**b**). Key anatomic landmarks: Native aortic valve annulus plane (*solid black arrow*), left main coronary emanating from left coronary sinus of Valsalva (*solid white arrow*), sinotubular junction (*dotted white arrow*), Edwards SAPIEN valve (*double-headed arrow*) and mitral valve annular calcifications (*). Note very high positioning of the Edwards SAPIEN valve 100 % above the native aortic valve annulus in contradiction to the conventional recommendation to implant the device 50 % below and 50 % above native aortic valve annulus

The exact definition of misplacement is specific to the TAVR device type. The optimal deployment location of the Medtronic CoreValve® device requires that the most inferior edge of the device, the inflow portion, should be positioned approximately 4–6 mm below the aortic annulus plane [61, 62] (Fig. 29). The optimal deployment location of the Edwards SAPIEN device is likely gained when 50 % of its height is below and 50 % of its height above the aortic annulus plane [10, 60]. (Fig. 30).

## Challenges and perspectives

TAVR is a novel procedure that provides unique opportunities for multimodality research comparing different imaging strategies and different THV devices. Given the exponential rise in the number of procedures being performed with varying local expertise, there is a need to standardise imaging protocols as part of pre- and post-procedure assessment of TAVR patients. Implementation of standardised protocols is crucial not only for precise assessment of the aortic valve apparatus and vascular access with different scanners in different institutions, but also for creating opportunities for further multicentre research. The SCCT expert consensus document devoted to this topic suggests potential algorithms and techniques for imaging before TAVR. Although there is growing evidence of the importance of MDCT in the pre-procedure assessment, one of the current challenges is to find the precise role of MDCT in valve assessment and sizing. Thus, developing a rational algorithm for imaging use both before and after implant should become a priority to avoid substantial redundancy in imaging. Another potential role for imaging is the assessment of outcome prediction, based on the diagnosis of both cardiac and non-cardiac comorbidities. If successful, this should allow for optimised patient selection for the currently costly procedure. Overall, current trends strongly suggest that imaging, in particular MDCT, will play an increasingly important role in all aspects related to the TAVR procedure.

## Summary

During the last decade transcatheter aortic valve replacement has become widely used in many centres across the world with good clinical outcomes. The planning and performance of the procedure are based on a multidisciplinary team approach, with imaging proving crucial for pre-procedure planning. MDCT plays an important role in assessing the aortic valve, aortic root and ascending aorta, as well as the access root for the procedure (i.e., ilio-femoral, direct aortic or subclavian) utilising the combination of multiplanar reconstruction and 3D imaging. TAVR technology and equipment are rapidly advancing, with increased utilisation of advanced MDCT imaging contributing to continuing outcome improvement and broadening of procedure applications.
